# Supplementary material for: Small RNA zippers lock miRNA molecules and block miRNA function in mammalian cells
Source: Nat Commun. 2017 Jan 3;8:13964. doi: 10.1038/ncomms13964 (PMC5216115; doi:10.1038/ncomms13964)
Supplement: Supplementary Information — Supplementary Figures [file ncomms13964-s1.pdf]

# Supplementary Information

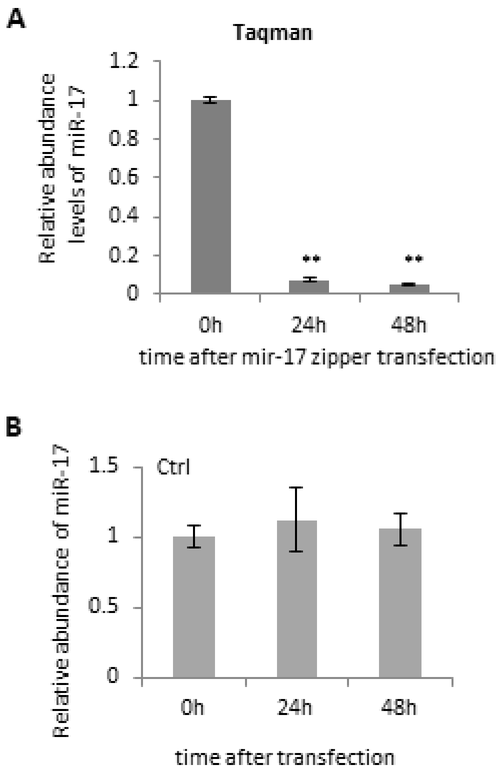


**Supplementary Figure 1**: A, Double confirmation of the knockdown of miR-17 expression by miR-17 zipper using Taqman technique for quantitative analysis of miRNA expression. B, Quantitative analysis indicating the negative control zipper does not affect the abundance of miR-17 in MDA-MB-231 cells. All analyses were repeated in triplicates. Data are mean ±SEM. **p<0.01 (*t*-test, n=3).

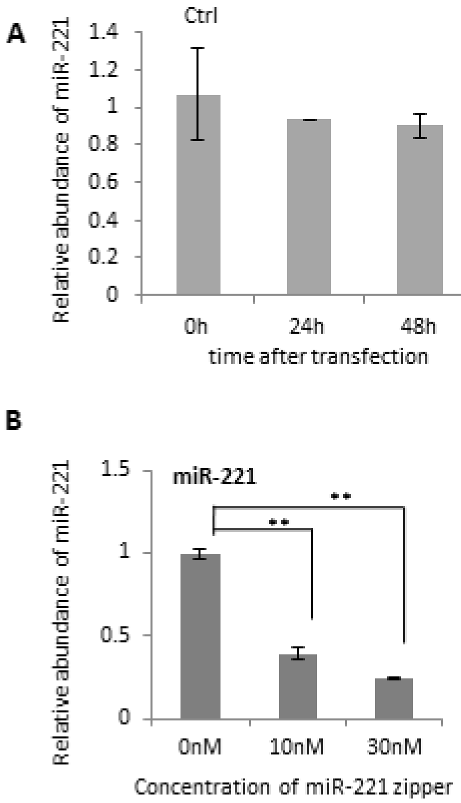


**Supplementary Figure 2**: A, Quantitative analysis indicating the negative control zipper does not affect the abundance of miR-221 in MDA-MB-231 cells. B, Dose response experiments were performed to determine the effect of miR-221 zipper to knockdown miR-221. All analyses were repeated in triplicates. Data are mean ±SEM. **p<0.01 (*t*-test, n=3).

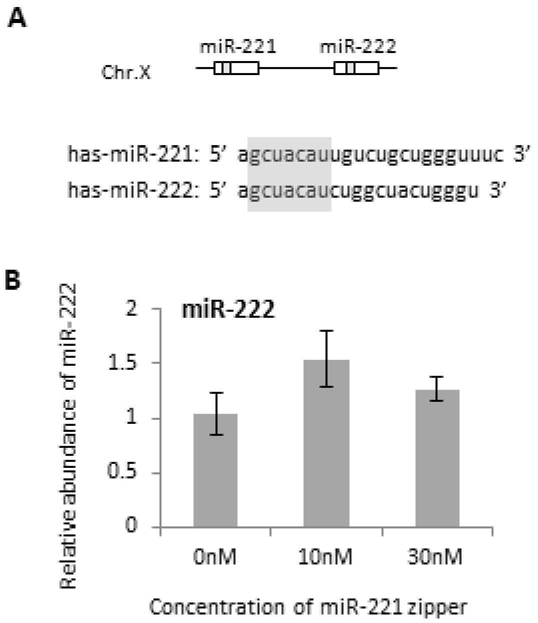


**Supplementary Figure 3:** A, The miR-221/222 cluster is located at Chromosome X, in which two miRNA members, miR-221 and miR-222, have different RNA sequence but sharing the same "seed" sequence as shown in grey shadow. B, Quantitative real-time PCR analysis showing the miR-222 levels did not change after transfection of miR-221 zipper into MDA-MB-231 cells.

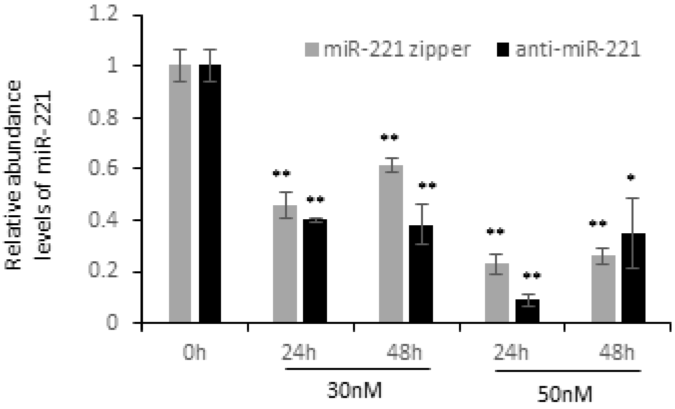


**Supplementary Figure 4**: A head to head comparison between miR-221 zipper and anti-miR-221, indicating both approaches can knockdown miR-221 abundance around 70-90% depending on the concentration and transfection efficiency. Data are mean ±SEM. *p<0.05, **p<0.01 (miR-221 zipper or anti-miR-221 groups *versus* 0h control groups, *t*-test, n=3).

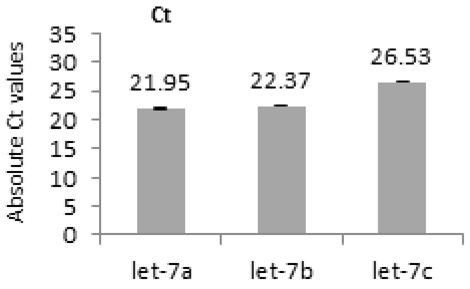


**Supplementary Figure 5**: The absolute Ct values of let-7a, let-7b and let-7c in MDA-MB-231 cells from a real-time PCR analysis. The experiment was repeated in triplicates. Data are mean + SEM (n=3).

A

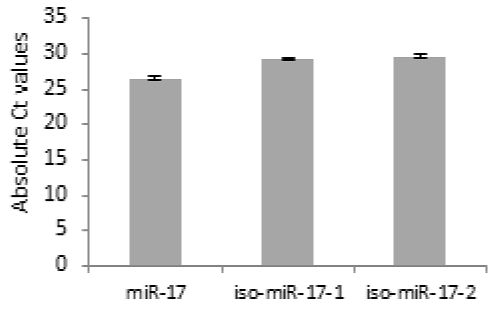


B

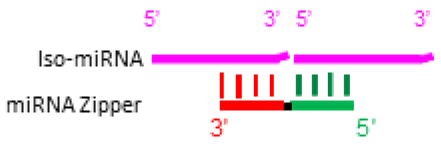


**Supplementary Figure 6**: A, The absolute Ct values of miR-17, iso-miR-17-1 and iso-miR-17-2 in MDA-MB-231 cells from a real-time PCR analysis. Data are mean + SEM (n=3). B, Schematic representation of the interaction between the miRNA zipper and iso-miRs.

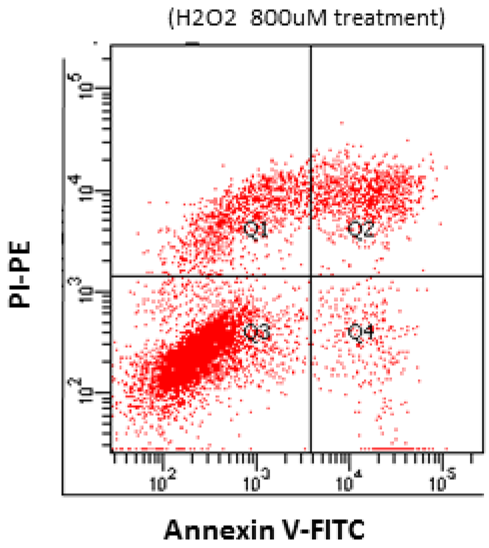


**Supplementary Figure 7**: Positive control for Annexin V staining of dead and apoptotic MDA-MB-231 cells treated by 800uM $H_2O_2$.

**A**

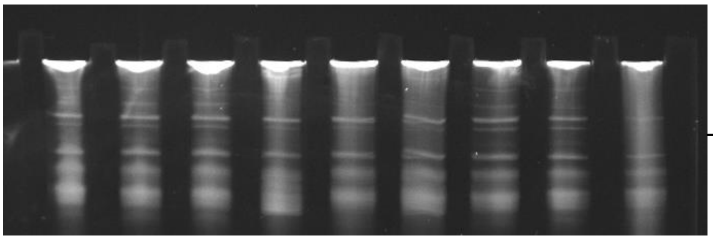


**B**

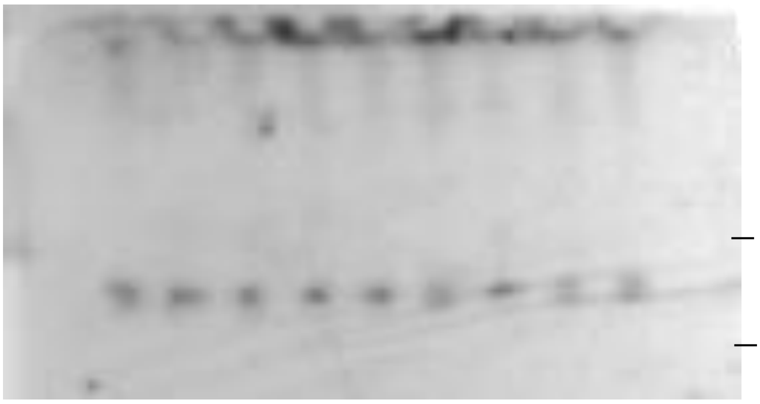


**Supplementary Figure 8:** Uncropped blots of Figure 3F. A: EB staining for tRNA. B: northern blot for miR-17.

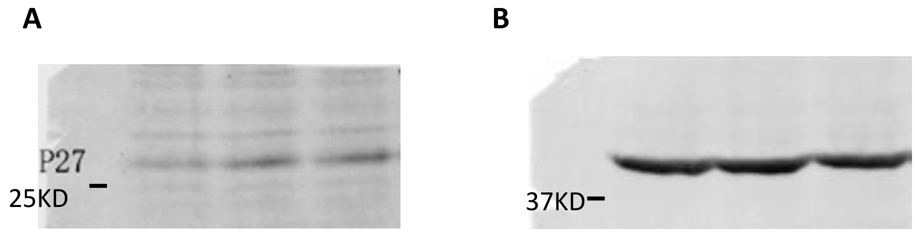


**Supplementary Figure 9:** Uncropped blots of Figure 6B. A: western blot for p27. B: western blot for β -actin.

A B

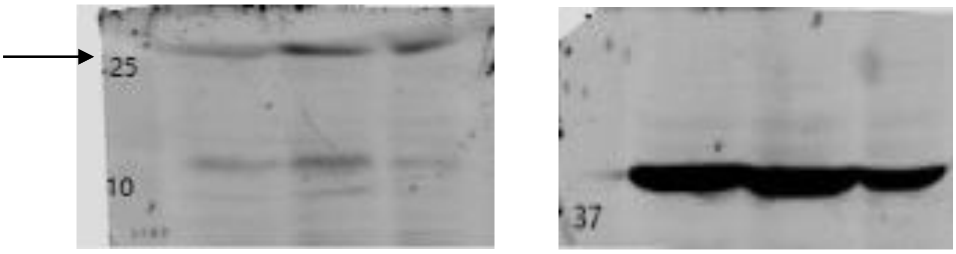


**Supplementary Figure 10:** Uncropped blots of Figure 6C. A: western blot for p27. B: western blot for β -actin.
